# Supplementary material for: The interactome of intact mitochondria by cross-linking mass spectrometry provides evidence for coexisting respiratory supercomplexes
Source: Mol Cell Proteomics. 2017 Dec 8;17(2):216–32. doi: 10.1074/mcp.RA117.000470 (PMC5795388; doi:10.1074/mcp.RA117.000470)
Supplement: Supplemental Data [file supp_RA117.000470_133922_0_supp_23978_fzffwf.zip › spectra_annotation/mito_spectra_annotation/163-1-1-1-10-1.pdf]

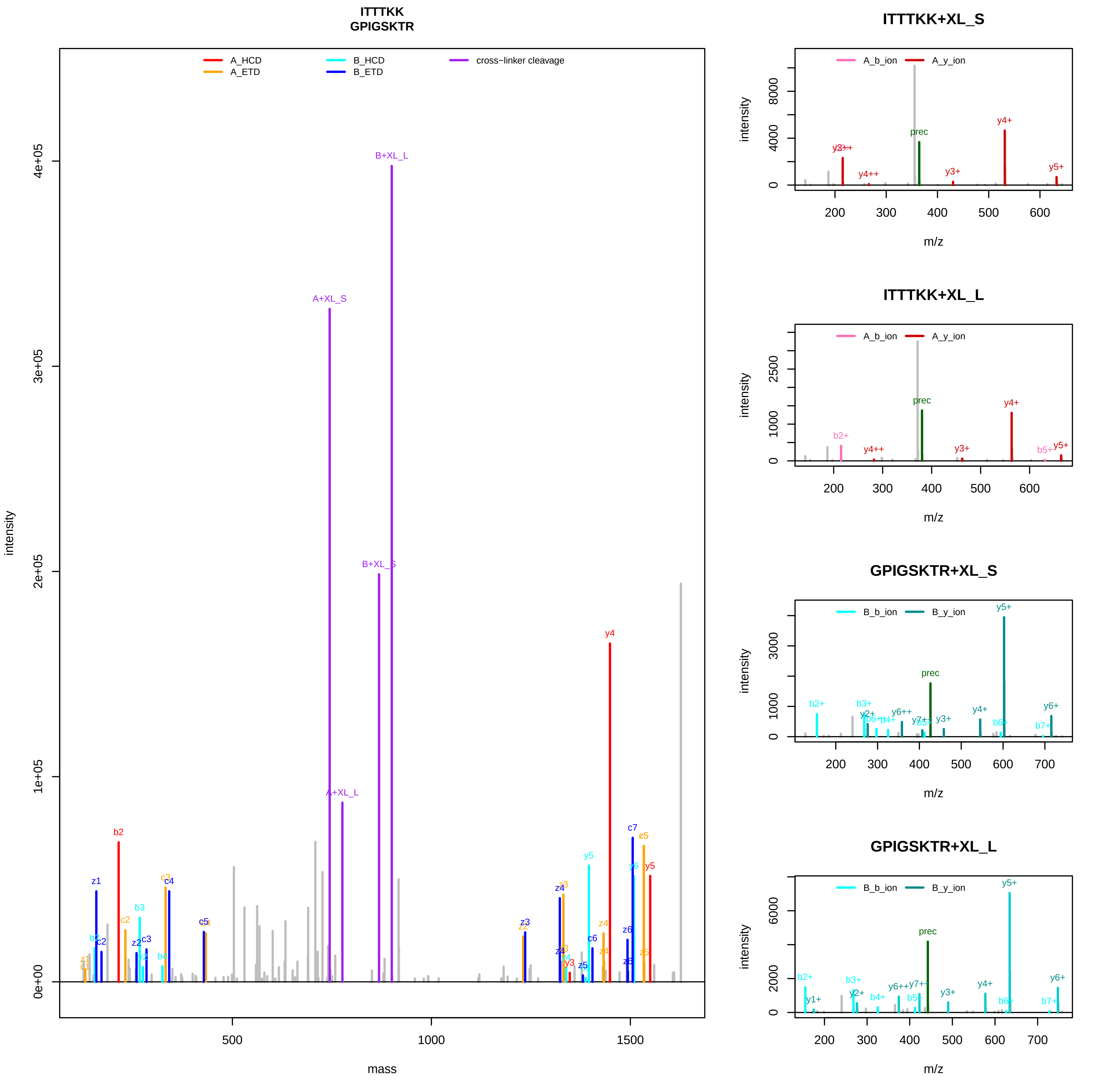
ITTTKK
GPIGSKTR
A_HCD
A_ETD
B_HCD
B_ETD
cross−linker cleavage
B+XL_L
A+XL_S
B+XL_S
A+XL_L
intensity
mass
0e+00
1e+05
2e+05
3e+05
4e+05
500
1000
1500
b2
z1
c2
b3
c2
z2
c3
c3
c4
b4
c5
z3
z2
z4
z3
y3
z5
c6
y5
z4
y4
z6
c7
y6
c5
y5
ITTTKK+XL_S
A_b_ion
A_y_ion
prec
y3++
y4++
y3+
y4+
y5+
ITTTKK+XL_L
b2+
b5+
GPIGSKTR+XL_S
B_b_ion
B_y_ion
b3+
y2+
b4+
y6++
y7++
b5+
y4+
b6+
b7+
y6+
GPIGSKTR+XL_L
y1+
m/z
